# Consequences of timing of organic enrichment provision on pig performance, health and stress resilience after weaning and regrouping

**DOI:** 10.1016/j.animal.2022.100637

**Published:** 2022-10

**Authors:** K. Bučková, R. Muns, J. Cerón, I. Kyriazakis

**Affiliations:** a Institute for Global Food Security, School of Biological Sciences, Queen's University Belfast, Belfast, BT9 5DL, UK; b Monogastric Research Group, Sustainable Agri-Food Sciences Division, Agri-Food and Bioscience Institute, Belfast, BT26 6DR, UK; c Interdisciplinary Laboratory of Clinical Analysis, Interlab-UMU, University of Murcia, 30100, Campus de Espinardo s/n, Murcia, Spain

**Keywords:** Enrichment, Finisher pigs, Regrouping, Resilience, Weaner pigs

## Abstract

Most pigs in slatted systems are provided with enrichment meeting only minimum legal requirements. We aimed to explore the effects of a novel enrichment treatment consisting of daily provided fodder beet and jute bags for pigs in slatted systems, and investigate the timing of enrichment provision on performance, health and stress resilience. We used 280 weaners allocated into standard (**S**, meeting only legal requirements consisting of a plastic toy and softwood) or enriched (**E**) treatment (n = 14 groups/treatment). At regrouping during the grower to finisher transition, pigs were either kept in the same treatment (**EE**, **SS**) or switched from enriched to standard (**ES**) and vice versa (**SE**); each treatment was replicated on five groups. Pigs were weighted at the start and end of weaner, and finisher stage, and feed intake was recorded. Occurrence of scouring, respiratory problems, locomotor disorders, tail, ear, and body lesions were recorded twice a week. Ten males per treatment were sampled for saliva on days 1, 2 and 4, either postweaning or after the housing switch. Saliva samples were analysed for cortisol, alpha-amylase, haptoglobin (**Hp**), and adenosine deaminase. Additionally, these pigs were sampled for hair at the start and end of weaner, and end of finisher stage to analyse for hair cortisol and cortisone. We found that E weaners consumed less feed (*P* = 0.04), had better FCR (**feed conversion ratio**, *P* = 0.03) and less ear lesions for two weeks postweaning (*P* = 0.04), and tended to have lower occurrence of scouring (*P* = 0.07) and higher salivary cortisol concentrations (*P* = 0.09) than S weaners. Effects of enrichment treatment during weaner stage on performance were carried through to finisher stage, with EE and ES pigs having better FCR (*P* = 0.0009) and higher BW (*P* = 0.0001) compared to SS and SE pigs. E treatment during finisher stage decreased feed intake (*P* = 0.04) and tended to decrease Hp levels (*P* = 0.07). There was a significant interaction between enrichment treatments during weaner and finisher stages on finisher body lesions: EE finishers had less lesions than SS, ES, and SE finishers (*P* = 0.04). There were no other significant differences caused either by enrichment treatment during weaner/finisher stage or their interaction. We conclude that the novel enrichment applied at weaner stage had positive effects on ear lesions and performance, which were carried through to finisher stage. Body lesions were affected by its application during both stages, with finishers receiving the enrichment treatment throughout (EE) having reduced body lesions than the rest of the finishers.

## Highlights

• We tested fodder beet and jute bags as novel enrichment for pigs in slatted systems.
• We investigated if enrichment mattered more when offered at weaner or finisher stage.
• Weaner enrichment improved ear lesions and performance at both stages.
• Enrichment provision throughout reduced finisher body lesions.
• Fodder beet and jute bags could be feasible enrichment for pigs in slatted systems.

## Implications

There is a need to find efficient and suitable enrichment for pigs in slatted systems. We tested fodder beet in combination with jute bags as novel enrichment for pigs in slatted systems and investigated at which stage of growth additional enrichment provision was more important. We found positive effects of novel enrichment during weaner stage on ear lesions and performance during both weaner and finisher stages. Additional enrichment during finisher stage alone was associated with less positive effects, but its provision throughout reduced body lesions.

## Introduction

Under natural conditions, pigs spend a large part of their active time searching for food by exploring their surroundings. They are motivated to perform behaviours such as rooting, even though their immediate needs are met, e.g., if they have sufficient quantities of food (Studnitz et al., 2007). According to European Union (**EU**) legislation, farmed pigs must have permanent access to a sufficient quantity of material to enable proper manipulation and investigation (EU, 2008). The material should be edible or feed-like, chewable, investigable and manipulable (EU, 2016). Most pigs housed in commercial slatted systems are provided with enrichment meeting only minimum legal requirements, which is often unsuitable or insufficient (Buijs and Muns, 2019, van de Weerd and Ison, 2019). One reason may be that there is no enrichment generally regarded as functional for pigs housed in predominating slatted systems (van de Weerd and Day, 2009, Chou et al., 2019).

Studies across species show that housing animals in environment with insufficient enrichment, i.e., with limited opportunities to fulfil natural behaviours, impairs their welfare (Vitalo et al., 2012, Zhang et al., 2021, Fureix et al., 2022). In pigs, barren housing has been found to cause negative affective states, increase occurrence of undesirable behaviours (e.g., tail biting), decrease inactivity, and impair immunity (Bolhuis et al., 2005, Douglas et al., 2012, Telkänranta et al., 2014, van Dixhoorn et al., 2016). Moreover, some studies have found negative effects of barren housing on pig growth, feed intake and meat quality (Beattie et al., 2000, Luo et al., 2020b).

The question at what stage of life enrichment should be provided is an active one. A range of different housing and rearing practices are used for pigs, some of which involve substantial changes in environment; e.g., outdoor reared pigs being finished inside, or straw use in the farrowing and/or weaner house, but subsequent housing is in fully- or part-slatted systems until slaughter (Day et al., 2002, Douglas et al., 2012). It was found that pigs switched from barren to enriched housing compensate the lack of opportunities in the previous housing by temporal increasing of some behaviours, e.g., play (Luo et al., 2020b). However, the results on switching pigs from enriched to barren housing are inconclusive. Loss of enrichment impairs affective states (Bolhuis et al., 2006, Douglas et al., 2012) and may increase undesirable behaviours, e.g., oral manipulation of pen mates (Luo et al., 2020b) or tail biting (Munsterhjelm et al., 2009), but experience with enrichment provision may be beneficial in the long-term, i.e., when housed without enrichment later in the life (Day et al., 2002, Telkänranta et al., 2014, Luo et al., 2020a). Day et al. (2002) found that when pigs had no prior experience of straw, tail biting was elevated for three weeks after being moved into grower/finisher accommodation. Similarly, Telkänranta et al. (2014) found that pigs with the provision of sisal ropes and paper before a housing switch had less severe tail damage later in life.

We aimed to test the effect of root vegetables and jute bags as functional enrichment for pigs housed in slatted systems, which cannot easily accommodate straw provision. Root vegetables are recommended enrichment for pigs (EU, 2016), due to their potential attractivity, as they are edible. Moreover, organic enrichment may have a beneficial effect on the development of gut microbiota and their influence on immune competence (Wen et al., 2021). However, it has yet to be experimentally tested. We also aimed to investigate the effect of the timing of enrichment provision. We hypothesised that the additional enrichment would improve: (1) performance and health of pigs during grower and finisher stage, and (2) chronic and acute stress response to weaning and subsequent litter mixing, as well as housing switch and regrouping during the grower to finisher transition. Furthermore, we predicted that switching pigs from enriched to barren housing would impair their short-term welfare, (i.e., increase stress response to housing switch), but there will be long-term benefits of the enrichment on finisher performance, health, and chronic stress response.

## Material and methods

### Animals and experimental design

The experiment was carried out at the experimental farm of the Agri-Food and Biosciences Institute (AFBI), Hillsborough, Northern Ireland from March to July 2021. We focused on the weaner – grower (42 days) and finisher stage (76 days) of rearing. For the experiment, we used two batches, each with 140 Duroc × (Large White × Landrace) pigs weaned at four weeks of age; batches 1 and 2 were separated by 3 weeks. Pigs in batches 1 and 2 originated from 12 and 15 l, respectively. The only selection criterion for piglets was good health (no sign of illness/health disorder) and BW ≥ 6 kg at weaning. All pigs were tail docked, with approximately 50 % of the tail removed within 24 h of birth.

At the beginning of the weaner stage, newly weaned pigs were allocated into a standard (**S**) or enriched (**E**) housing. S enrichment met minimal legal requirements (EU, 2008) and consisted of a plastic biting toy Porcichew (15 cm in a diameter; Nutrapet ltd., U.K.) and a piece of softwood (30 cm × 4.5 × 4.5 cm) suspended on a chain. E pigs had additional access to fodder beet in a rack (mounted at a height of 0.3 m) and suspended jute bags (73 × 33.5 cm). Each pen was provided with 1.5–2.5 kg of beet per day (1.88 ± 0.4 kg, mean ± SD). Fresh beet was provided for two weeks postweaning and previously preserved beet over the remaining period. If there were any leftovers on the racks, they were reused. One jute bag was provided per pen and replaced when almost consumed. Jute bags have been found to have positive effect on damaging behaviours (Ursinus et al., 2014, Buijs and Muns, 2019) and are recommended enrichment for pigs in slatted housing systems (EU, 2016).

Each treatment was replicated on 14 pens (10 weaners/group). Each group was balanced for BW and gender. All pigs were housed in the same building with six rooms, and each room contained six pens. Each pen measured 2.7 × 1.4 m. Pens were assigned to a treatment randomly, while ensuring to have seven enriched pens per batch distributed across three rooms. The rooms and pens did not differ in any variables, which could have confounded the results (temperature, layout etc.). Pens had plastic fully slatted floor and non-transparent walls. Feed was delivered via a multispace dry feeder taking 0.23 m^2^ (Etra Feeders, Northern Ireland) and water via a bowl drinker with a nipple inside (Echberg Drik-O-Mat, Denmark).

On day 42 postweaning, i.e., at the beginning of grower – finisher stage, pigs were transported to a finishing unit located approximately 1 km from the previous accommodation. This part of the study was designed as a 2 × 2, i.e., where pigs were kept either in the same enrichment treatment (**SS** and **EE** treatments) or were switched from enriched to standard (**ES**) and vice versa (**SE**) enrichment treatments. Enrichment for SS and ES finishers consisted of the same items as for weaners, but the amount was doubled, i.e., two suspended toys and two pieces of softwood were provided per pen. EE and SE pigs in addition had access to fodder beet in a rack (mounted at a height of 0.5 m) and two suspended jute bags. Each pen was provided with 2.5–3.5 kg of beet per day (3.12 ± 0.21 kg, mean ± SD) for 41 days and 3.5–4.5 kg (4.12 ± 0.21 kg, mean ± SD) for the remaining period. Beet offered to finishers was previously stored in a freezer but was defrosted before being offered to pigs. If there were any leftovers on the racks, they were reused. Two suspended jute bags per pen were provided and replaced when almost consumed.

Each treatment was replicated on five groups. Each group consisted of 14 finishers and was balanced for previous group, BW, and gender. Both experimental batches were housed in two houses (called House 2 and House 3) within the same building. Pigs from batch 1 were housed in 10 pens of the same room of House 3. Five pens were equipped with a single space dry feeder taking 0.114 m^2^^,^ and five pens were equipped with a single space wet feeder taking 0.48 m^2^ (Etra Feeders, Northern Ireland). A pen measured either 3.25 × 2.00 m (if equipped with a dry feeder) or 3.30 × 3.90 m (if equipped with a wet feeder). Pigs from batch 2 were housed in two rooms (five pens per room) of House 2. A pen measured 3.3 × 3.9 m. All pens in House 2 were equipped with a MLP pro feeder (Schauer®, Austria), each taking 1.43 m^2^. Pens in both houses had a wall nipple drinker, concrete fully slatted floor. Pens were assigned to treatment randomly but ensuring to have 2/3 pens per treatment per batch. Finishers were enrolled in the experiment for 76 days (i.e., until transported to a slaughterhouse). Thus, the total length of the experimental period per batch was 118 days. Feed and water were provided *ad libitum* during both stages, with all pigs receiving the same diets. Weaners were fed the following commercial diets: starter 1 (∼12 days), starter 2 (∼8 days), link (7 days) and grower diet (∼15 days). Finishers were fed commercial grower (15 days) and finisher diet (62 days). Enrichment treatments are shown in Supplementary Fig. S1-4.

### Data sampling

#### Performance

During the weaner stage, feed intake was measured at a pen level at the end of each diet offered. During the finisher stage, feed intake was measured daily at a pen (over 73 days) and individual level (over 77 days) for batch 1 and batch 2, respectively. Beet consumption was measured every morning (i.e., before fresh provision was made) by weighing leftovers. Pigs were weighted three days preweaning, on day 40 postweaning (i.e., two days before housing switch) and 76 days after housing switch (at the end of the finisher stage).

#### Health

Pig health was assessed twice a week (mostly Mondays and Fridays), which resulted in 22 observations per batch. The health scoring system was based on Welfare Quality assessment protocol (Welfare Quality®, 2009) and is presented in Table 1. Scouring and respiratory problems (coughing, sneezing) were assessed at a group level. Pigs were individually assessed for locomotor disorders (bursitis, lameness), and tail, ear and body lesions. Only the outer side of one ear was scored for ear lesions. Only one side of the pig was scored for body lesions (excluding ears and tails, as they were assessed separately). Health was assessed by the same person standing in front of a pen. Pens were entered only if necessary (e.g., when a pig was lying behind others and could not be assessed otherwise). Most parameters were recorded as present/absent (1/0). Respiratory problems were recorded as a number of coughs and sneezes per observation.

**Table 1:** Health scoring system used to assess weaner and finisher pigs, based on the Welfare Quality assessment protocol (Welfare Quality®, 2009).

Health parameter	Scoring level	Score	Score description
Scouring	Group	0	No liquid faeces visible on the floor
		1	Liquid faeces visible on the floor
Respiratory problems	Group		
Coughing			Number of coughs recorded per observation
Sneezing			Number of sneezes recorded per observation
Locomotor disorders	Individual		
Bursitis		0	No evidence of bursae
		1	At least one bursa present
Lameness		0	Normal gait
		1	Difficulty in walking
Tail lesions	Individual	0	No evidence of tail biting
		1	Any sign of tail biting present
Ear lesions	Individual	0	Undamaged ears/lesions with no sign of blood
		1	Blood is visible on outer side of ear
Body lesions	Individual	0	No visible skin injuries/up to 4 lesions visible on the pig body (1 lesion = 2 cm or longer)
		1	More than 4 lesions were found on the pig body

#### Stress resilience

One male pig per pen (10 sampled pens/treatment) chosen randomly was sampled for saliva one day preweaning and on days 1, 2, 4 and 40 postweaning. Days 1, 2 and 4 were chosen to assess the effect of the enrichment on acute stress response to weaning and mixing. Saliva samples were analysed for levels of cortisol and alpha-amylase as biomarkers of stress, haptoglobin (**Hp**) as a biomarker of inflammation, and adenosine deaminase (**ADA**) as an immune system biomarker. The same animals were sampled at finisher stage 1, 2 and 4 days after the housing switch. These days were chosen to assess acute stress response to transport, housing switch and regrouping. Detailed description of the saliva sampling procedure is provided in the Supplementary Material S1. In all cases, saliva was sampled on a synthetic swab attached to a plastic cable tie. We aimed to obtain a minimum 300 μl of pig saliva. The swab was thereafter inserted in a Salivette® Cortisol tube. The samples were kept on ice until all animals were sampled. Once completed, the samples were centrifuged (3 000 rpm, 10 min) and stored in a freezer at −80 °C until analysis. Cortisol concentration was measured by a solid-phase, competitive chemiluminescent enzyme immunoassay that uses a polyclonal rabbit anti-cortisol antibody (Immulite/Immulite 1 000 cortisol, Siemens Medical Solutions Diagnostics), previously validated for porcine saliva samples (Escribano et al., 2012). Salivary α-amylase activity was measured by a commercial method (a-Amylase, OSR6182, Beckman Coulter), previously validated in porcine saliva (Fuentes et al., 2011) in an automated analyser (Olympus AU600, Olympus Diagnostica). Hp was measured by an assay based on AlphaLisa (Perkin Ellmer) technology previously validated (Contreras-Aguilar et al., 2021). ADA was analysed with a commercially available spectrophotometric automated assay (Adenosine Deaminase assay kit, Diazyme Laboratories) previously validated for porcine saliva (Tecles et al., 2018). Total ADA was measured in the absence and presence of the specific ADA1 inhibitor erythro-9-(2-hydroxy-3-nonyl) adenine in the reaction media at a final concentration of 0.47 mM, as previously described (Contreras-Aguilar et al., 2020). ADA assay was carried out in an Olympus AU600 (Beckman Coulter Life Sciences) analyser.

On day 37, a hair sample from the same ten male weaners was taken to analyse cortisol and cortisone to measure chronic stress response. A sample which was used only as a covariate was obtained either a day preweaning (batch 2) or at weaning (batch 1). A hair sample was also taken 76 days after housing switch, i.e., at the end of finisher stage. Hair was shaved by an electric hair clipper from the rump in preweaning piglets. However, there was not enough re-grown hair on the rump at the end of weaner stage. Therefore, we took a hair sample from the right flank in weaners and finishers. Each hair sample was put in a plastic zip-lock bag and stored in a fridge at 8 °C until analysis. The protocol used for cortisol and cortisone extraction from hair was based on a previously described method (Davenport et al., 2006). The hair samples were weighed (60 ± 8.43 mg, mean ± SD) and given a colour score (0 = 100 % white,1 = 90 % white and 10 % brown, 2 = 75 % white and 25 % brown, 3 = 50 % white and 50 % brown) before being placed in a polypropylene tube and covered with isopropanol (5 ml). The tube was mixed at room temperature, centrifuged (1 500*g,* 1 min) and the isopropanol discarded. The samples were washed again with isopropanol and left at room temperature until completely dry. Next, the hair samples were cut into small pieces placed in tubes with balls and pulverised to a fine powder in a homogeniser (Precellys Evolution homogeniser, Bertin Technologies, France). The pulverised hair was incubated with 1 ml of methanol for 18 h at RT with continuous gentle agitation for steroid extraction. Samples were then centrifuged (2 000*g*, 5 min), and 0.6 ml of each methanol extract was aliquoted into a new Eppendorf tube. The samples were evaporated to dryness in a Speed Vac Concentrator (Concentrator 5301, Eppendorf). The dry extracts were reconstituted with 0.1 ml of phosphate buffer saline and stored at –80 °C until measuring in all samples (López-Arjona et al., 2020).

### Statistical analysis of results

Plots of predicted values against residuals and distribution histograms of residuals were visually inspected to check the homoscedasticity and normality assumptions of models and transformed if needed. Data were inspected for outliers by the boxplot method (Rusakovica et al., 2017). Any outliers were removed from the statistical models (if not specified, this was the reason for sample size reduction). The separate models were always run for each dependent variable as well as each stage of pig growth. A covariance structure in case of mixed models was chosen based on Akaike and Bayesian information criteria. If there was a significant effect of any independent variable, posthoc analyses were used to determine treatment differences (Tukey-Kramer test was applied). The same was done for statistical trends (0.1 > P > 0.05). If there was no significant effect or trend caused by enrichment treatment, numeric values were not reported. Non-significant effects of explanatory variables and the interaction between enrichment treatment and day/week were not reported. Batch (1/2) was included in all models, but it is not reported in any case, as this effect was not relevant to the objectives of the paper. Three-way interactions (enrichment treatment during weaner stage*enrichment treatment during finisher stage*time point) were not significant, so they were dropped from all models. Data were analysed in SAS® Studio.

#### Performance

The sums of total pen feed intake were calculated and then divided by the number of pigs in a pen, and the number of days to get the average daily feed intake (**ADFI**) per animal. ADFI was analysed by general linear models (PROC GLM in SAS). The dependent variable was ADFI (kg) per animal in a group. In the weaner model, enrichment treatment (S/E, n = 14 per treatment), and average pen preweaning BW were included as fixed effects. In the finisher model, enrichment treatment during weaner stage (S: n = 8, E: n = 9), enrichment treatment during finisher stage (S: n = 9, E: n = 8), their interaction, preweaning BW and BW measured 2 days before housing switch were included as fixed effects. The correlation between average pen preweaning BW and average pen BW measured 2 days before housing switch calculated by Pearson correlation coefficient was moderate (r = 0.44), so both BWs were included in the finisher model.

BW (kg) measured at the end of weaner and finisher stages were analysed by generalised linear mixed models (PROC MIXED in SAS). In the weaner model, enrichment treatment (S: n = 137, E: n = 139), preweaning BW and gender were included as fixed effects. The BW of one pig which was euthanised at the beginning of the study, as well as the BW of three other animals experiencing poor health, were not included in the analysis. In the finisher model, enrichment treatment during weaner stage (S: n = 133, E: n = 134), enrichment treatment during finisher stage (S: n = 132, E: n = 135), their interaction, BW measured 2 days before housing switch, and gender were included as fixed effects. Thirteen pigs were not included in the analysis as they did not remain in the study until the end of the finisher stage (for detailed information, see the Attrition rate section). The correlation between preweaning BW and BW measured 2 days before housing switch calculated by Pearson correlation coefficient was high (r = 0.69). Therefore, only BW measured 2 days before the housing switch was included in the finisher model. Pig nested within a group was a random effect in both above models.

Weaner feed conversion ratio (**FCR**) was calculated as the total amount of feed eaten per animal divided by BW gain (BW measured 2 days before housing switch minus preweaning BW). FCR (log transformed) was analysed by generalised linear mixed models. In the weaner model, enrichment treatment (S: n = 135, E: n = 136) and gender were included as fixed effects. FCR of finishers was calculated as total amount of feed eaten per animal during finisher stage divided by BW gain (final BW minus BW measured 2 days before housing switch). In the finisher model, enrichment treatment during the weaner stage (S: n = 129, E: n = 128), enrichment treatment during the finisher stage (S: n = 127, E: n = 130), their interaction, and gender were included as fixed effects. Pig nested within a group was a random effect in both above models. The same pigs excluded for the BW analysis were not included for the FCR analysis.

#### Health

Health parameters were analysed at a group level, i.e., summed for each pen. Sums were further calculated for three time periods in weaners (weeks 1–2, 3–4, 5–6), and four in finishers (weeks 1–2, 3–5, 6–8, 9–11). Data were analysed by repeated measures mixed models (PROC MIXED with repeated statement in SAS). Data on finisher body lesions achieved normal distribution after square root transformation, but other health measurements did not. Therefore, PROC GLIMMIX with Poisson distribution and log link was used to analyse these variables. In weaners, respiratory problems, locomotor disorders and tail lesions were recorded rarely so they were not statistically analysed. After removing outliers, scouring and body lesions were not present in weaners during periods 3–4 and 5–6 weeks, respectively, and these time periods were not included in the analysis. The final weaner data set consisted of values obtained from 11 to 14 groups per treatment per week period. Number of excluded values for weaner health parameters is given in Supplementary Table S1. In the weaner models, enrichment treatment, week, and their interaction were included as fixed effects. Period was included as a repeated measure. After removing outliers, tail lesions were not present in finishers during weeks 1–2. Therefore, this period was not included in the model. For finishers, enrichment treatment during weaner stage, enrichment treatment during finisher stage, week, the interaction between them, and the interaction between enrichment treatment during weaner/finisher stage and week were included as fixed effects. Period was included as a repeated measure. The final finisher data set consisted of values obtained from 9 to 10 groups per treatment per week period. Number of excluded values for finisher health parameters is given in Supplementary Table S2.

#### Salivary and hair biomarkers

Concentrations of cortisol (μg/dl), salivary alpha-amylase (IU/l), Hp (IU/l), and ADA (IU/l) were analysed by repeated measures mixed models. Weaner amylase values were square root transformed before analysis. Finisher cortisol, alpha-amylase and Hp values were log transformed before analysis. In the weaner models, the fixed effects were enrichment treatment, day (1/2/4), preweaning BW, preweaning concentration of the marker, and the interaction between enrichment treatment and day. Day was included as a repeated measure. The final data set consisted of values obtained from 9 to 10 weaners per treatment per day. Number of missing/excluded values for weaner salivary biomarkers is given in Supplementary Table S3. Preliminary analysis revealed that there was no significant effect of preweaning salivary cortisol concentrations on later cortisol levels, so this fixed effect was dropped from the final weaner model. In the finisher models, the fixed effects were enrichment treatment during weaner stage, enrichment treatment during finisher stage, their interaction, day (1/2/4), the concentration of the biomarker measured at the end of weaner stage and the interactions between day and enrichment treatment during weaner/finisher stage. Day was included as a repeated measure. The correlation between preweaning BW and BW measured 2 days before housing switch calculated by Pearson correlation coefficient was weak (the correlation for cortisol: r = 0.30, alpha-amylase: r = 0.31, Hp: r = 0.26, ADA: r = 0.27), so both BWs were included as fixed effects. The correlation between preweaning cortisol, alpha-amylase, and Hp concentrations and those measured before housing switch were high (cortisol: r = 0.81, alpha-amylase: r = 0.63, Hp: r = 0.69), so we included only the latter concentration. The correlation between preweaning ADA concentration and the concentration measured before housing switch was moderate (r = 0.48), so both covariates were included in the model. The final data set consisted of values measured in 7–10 finishers per treatment per day. Number of excluded values for finisher salivary biomarkers is given in Supplementary Table S4.

Hair cortisol and corticosterone concentrations (pg/mg) from samples taken on day 37 postweaning and 76 days after housing switch were analysed by general linear models. In the weaner cortisol model, the fixed effects were enrichment treatment (S: n = 9, E = 10), preweaning BW, preweaning hair cortisol concentration and colour score (0/1/2/3). In the weaner cortisone model, the fixed effects were enrichment treatment (S: n = 9, E = 10), preweaning BW, preweaning hair cortisone concentration and colour score. In the finisher cortisol model, the fixed effects were enrichment treatment during weaner stage (S: n = 9, E = 10), enrichment treatment during finisher stage (S: n = 10, E = 9), their interaction, hair cortisol concentrations measured on day 37, and colour score. The correlation between preweaning BW and BW measured 2 days before regrouping calculated by Pearson correlation coefficient was weak (r = 0.19). Therefore, both BWs were included in the finisher hair cortisol model as fixed effects. The correlation between hair cortisol before weaning and regrouping was high (r = 0.97), so we included only the latter one in the analysis. In the finisher cortisone model, the fixed effects were enrichment treatment during weaner stage (S: n = 10, E = 8), enrichment treatment during finisher stage (S: n = 9, E = 9), their interaction, hair cortisone concentrations measured on day 37, and colour score. The correlation between preweaning BW and BW measured 2 days before regrouping calculated by Pearson correlation coefficient was weak (r = 0.21), so both BWs were included in the finisher hair cortisone model as fixed effects. The correlation between hair cortisone concentration before weaning and regrouping was also weak (r = 0.26), so both concentrations were included in the analysis.

## Results

### Attrition rate

One S pig was euthanised at the beginning of the study due to lameness. Furthermore, one lame E pig was not transported to the finishing unit, four pigs died during the finisher stage (two SS and two ES), and seven finishers were euthanised due to health disorders during the finisher stage (two SS, one EE, one ES, three SE). This resulted in 65 SS, 68 EE, 67 ES, and 67 SE finishers.

### Performance

The results on weaner and finisher performance are presented in Table 2. In weaners, enrichment treatment had a significant effect on ADFI, as S pigs consumed more feed than pigs on E (F_1,24_ = 4.72, *P* = 0.04; Table 2). Beet consumption was not statistically analysed, but average daily pen intake was 1.72 ± 0.63 kg (mean ± SD). Enrichment treatment did not significantly affect weaner BW measured before the housing switch, but weaners, which were heavier before weaning, were also heavier before the housing switch (F_1,270_ = 337.11, *P* < 0.0001). Enrichment treatment had a significant effect on FCR during weaner stage, as E weaners had improved FCR compared to those housed on S (F_1,267_ = 4.86, *P* = 0.03).

**Table 2:** The effect of enrichment treatment on average daily feed intake (ADFI), BW and feed conversion ratio (FCR) in weaner and finisher pigs. Data are presented as LS means with SEM. Weaner pigs received standard (S) or enriched (E) treatments. Finisher pigs were kept either in the same enrichment treatment (SS and EE treatments) or were switched from enriched to standard (ES) and vice versa (SE) enrichment treatments.

	Housing during weaner stage				Housing during finisher stage					
Parameter	S	E	SEM	P-value	SS	EE	ES	SE	SEM	P-value^2^
ADFI (kg/animal)	0.85^a^	0.82^b^	0.01	0.04	2.71^a^	2.62^b^	2.75^a^	2.60^b^	0.04	0.04
BW (kg)	33.7	33.4	0.36	0.49	117^a^	119^b^	121^b^	116^a^	0.60	0.0001
FCR^1^ (Total feed intake, kg/BW gain, kg)	1.50^a^ (1.47–1.53)	1.44^b^ (1.41–1.48)		0.03	2.39^a^ (2.33–2.45)	2.30^b^ (2.24–2.36)	2.30^b^ (2.24–2.37)	2.43^a^ (2.34–2.52)		0.0009

a-b Values within a row with different superscripts differ significantly at *P* < 0.05, but they apply only to comparisons within weaner and within finisher stages.

1 Data were analysed after transformation by natural logarithms and are presented as back-transformed means with confidence intervals.

2 Finisher P-value represents either effect of the enrichment treatment during finisher stage (ADFI) or the effect of enrichment treatment during weaner stage (BW, FCR). There were no other significant differences or trends caused by enrichment treatment during weaner/finisher stage or their interaction.

In finishers, there was no significant effect of enrichment treatment during weaner stage or the interaction between enrichment treatment during weaner and finisher stages on ADFI. However, there was a significant effect of enrichment treatment on ADFI during finisher stage, as S pigs consumed more feed than pigs on E (F_1,10_ = 5.64, *P* = 0.04). Beet average daily pen intake was 2.99 ± 0.64 kg (mean ± SD) for 41 days and 3.55 ± 0.76 kg (mean ± SD) for the remaining finisher period.

Neither enrichment treatment during the finisher stage, nor the interaction between housing during weaner and finisher stages affected BW measured at the end of finisher. Enrichment treatment during the weaner stage had a significant effect on BW at the end of finisher stage, as EE and ES pigs were heavier than SS and SE pigs (F_1,260_ = 15.32, *P* = 0.0001). There was a significant effect of gender and initial BW on finisher BW. Males and pigs with higher initial BW were heavier (gender: F_1,260_ = 58.39, initial BW: F_1,260_ = 443.22; *P* < 0.0001). Neither enrichment treatment during finisher stage nor the interaction between weaner and finisher enrichment treatment significantly affected finisher FCR. Enrichment treatment during weaner stage had a significant effect on FCR, as EE and ES finishers had better FCR than SS and SE finishers (F_1,251_ = 11.35, *P* = 0.0009). Males had better FCR (gender: F_1,251_ = 35.62, *P* < 0.0001).

### Health variables

**Fig. 1:**
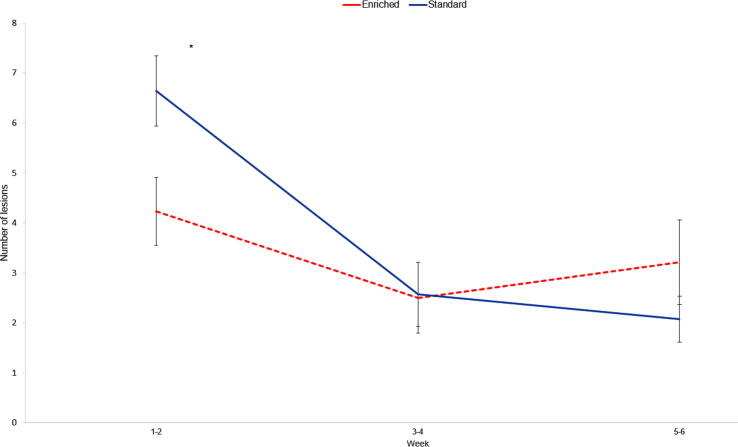
Effect of enrichment treatment and week interaction on number of lesions scored for six weeks postweaning in pigs receiving different enrichment treatments. Enriched pigs had decreased (*P* < 0.05) occurrence of ear lesions for two weeks postweaning than standardly housed pigs, indicated by an asterisk. Standard treatment: Blue line. Enriched treatment: Red dashed line. Error bars: SEM.

E weaners tended to have a decreased occurrence of scouring compared to S pigs (F_1,23_ = 3.74, *P* = 0.07), but there was no significant effect of enrichment treatment on ear or body lesions. However, the interaction between enrichment treatment and week tended to be significant for ear lesions (F_2,51_ = 3.05, *P* = 0.06). Posthoc analysis revealed that E weaners had less ear lesions than S weaners during weeks 1–2 (*P* = 0.04, Fig. 1). There was a significant effect of week on ear and body lesions (ear lesions: F_2,51_ = 11.55, *P* < 0.0001; body lesions: F_1,23_ = 8.21, *P* = 0.009). Ear lesions occurred more in weeks 1–2 compared to weeks 3–4 and 5–6, and body lesions occurred more in weeks 1–2 than in weeks 5–6.

**Fig. 2:**
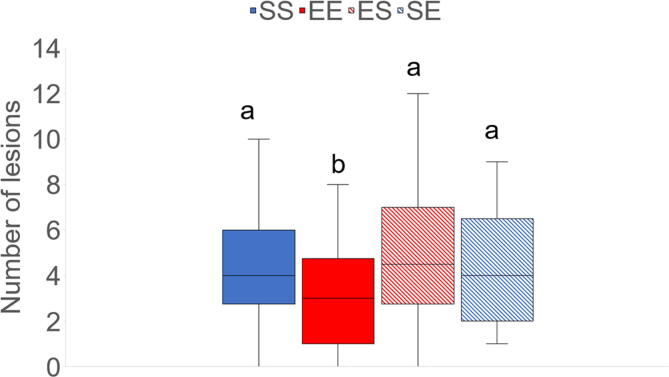
Effect of the interaction between enrichment treatment during the weaner and finisher stages on number of body lesions scored over 11 weeks. Weaner pigs received standard (S) or enriched (E) treatments. Finisher pigs were kept either in the same enrichment treatment (SS and EE treatments) or were switched from enriched to standard (ES) and vice versa (SE) enrichment treatments. ^a, b^ Boxes with different superscripts differ significantly at *P* < 0.05.

In finishers, enrichment treatment either during weaner or finisher stage did not have significant effect on scouring. However, there was a significant effect of week (F_3,49_ = 4.41, *P* = 0.01): scouring occurred less in weeks 1–2 compared to weeks 3–5, and tended to occur less in weeks 1–2 than in weeks 5–6. It also occurred less in weeks 3–5 compared to weeks 9–11. There was no significant effect of enrichment treatment on respiratory problems. However, there was a significant effect of week (F_3,48_ = 4.99, *P* = 0.004): respiratory problems occurred less in weeks 1–2 than in weeks 3–5 and 9–11. They also occurred less in weeks 6–8 than in weeks 3–5 and 9–11. Similarly, enrichment treatment did not significantly affect locomotor disorders, but there was a significant effect of week (F_3,50_ = 7.29, *P* = 0.0004): the occurrence of locomotor disorders was less frequent in weeks 1–2 than in weeks 3–5, 6–8 and 9–11. Locomotor disorders were also less frequent in weeks 6–8 than 9–11 and tended to be less frequent in weeks 3–5 compared to weeks 9–11. There was no significant effect of enrichment treatment on tail lesions. However, there was a significant effect of week (F_2,34_ = 22.55, *P* < 0.0001): tail lesions were less frequent in weeks 3–5 than in weeks 6–8 and 9–11. Moreover, they occurred less in weeks 6–8 than in weeks 9–11. There was no significant effect of enrichment treatment on ear lesions, but there was a significant effect of week (F_3,49_ = 64.03, *P* < 0.0001): finishers had more ear lesions in weeks 1–2 than in the rest of the weeks. They also had more ear lesions in weeks 9–11 than in weeks 3–5 and 6–8. Enrichment treatment during weaner and finisher stages did not have a significant effect on body lesions. However, the interaction between enrichment treatment during weaner and finisher stages was significant (F_1,15_ = 4.96, *P* = 0.04), which was caused by the decreased occurrence of body lesions in EE pigs compared to SS (*P* = 0.05), ES (*P* = 0.02) and SE (*P* = 0.01) pigs (Fig. 2). There was also a significant effect of week (F_3,46_ = 17.65, *P* < 0.0001). Finishers had decreased occurrence of body lesions in weeks 3–5 compared to weeks 1–2, 6–8 and 9–11.

### Salivary and hair biomarkers

**Fig. 3:**
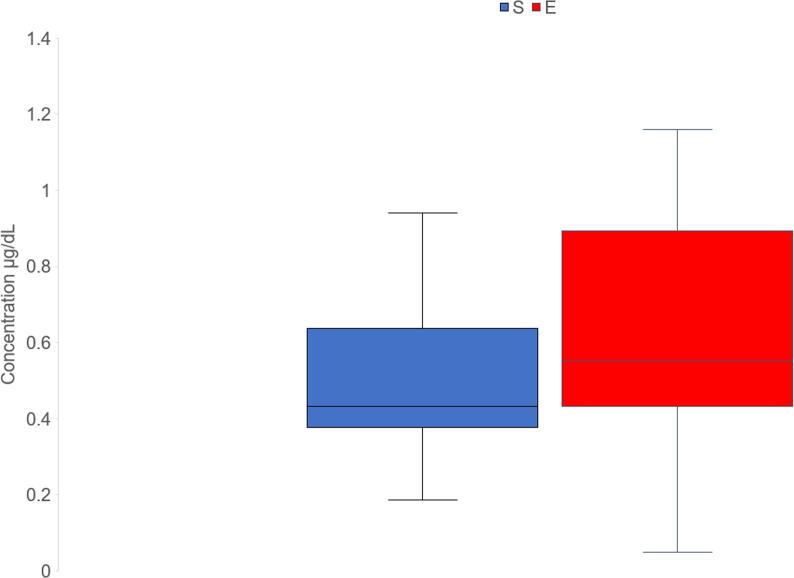
Effect of enrichment treatment during weaner stage on salivary cortisol concentrations after weaning. Enriched pigs (E) tended (*P* < 0.1) to have increased cortisol concentrations compared to standardly housed pigs (S).

**Fig. 4:**
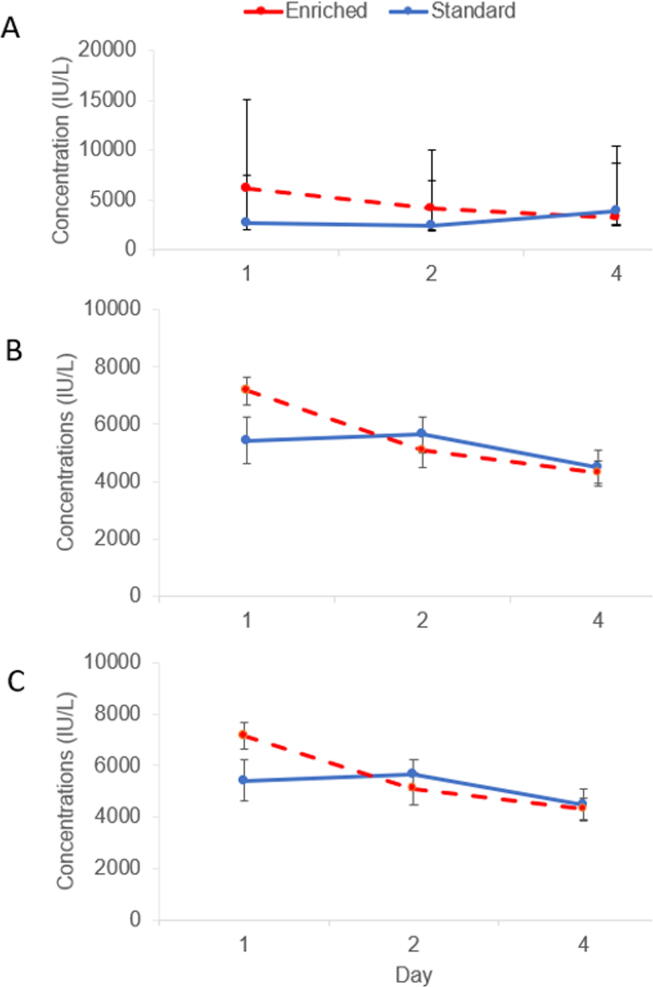
Effect of enrichment treatment and day interaction on alpha-amylase (A), haptoglobin (B), and adenosine deaminase (C) concentrations (IU/l) measured on days 1, 2, and 4 in pigs postweaning receiving different enrichment treatments. Enriched pigs tended (*P* < 0.1) to have higher alpha-amylase, haptoglobin and adenosine deaminase concentrations than standardly housed pigs on day 1. Alpha-amylase concentrations of enriched pigs also tended to be higher on day 2. Standard treatment: Blue line. Enriched treatment: Red dashed line. Error bars: SEM (Haptoglobin, adenosine deaminase)/ Confidence intervals (Alpha-amylase).

In weaners, enrichment treatment tended to have a significant effect on salivary cortisol concentrations. E weaners tended to have higher salivary cortisol concentrations than S pigs (F_1,15_ = 3.23, *P* = 0.09; Fig. 3). Pigs which were heavier preweaning had higher salivary cortisol concentrations (F_1,15_ = 5.22, *P* = 0.04). Enrichment treatment did not have a significant effect either on hair cortisol or cortisone concentrations at the end of weaner stage. There was no significant effect of enrichment treatment on weaner alpha-amylase concentrations, but the interaction between enrichment treatment and day tended to be significant (F_2,31_ = 2.59, *P* = 0.09; Fig. 4A). On day 1 and day 2, E pigs tended to have higher amylase concentrations compared to S pigs on day 1 and day 2. There was no significant effect of enrichment treatment on weaner Hp concentrations. However, the interaction between enrichment treatment and day tended to be significant (F_2,35_ = 2.68, *P* = 0.08): E weaners tended to have higher Hp concentrations than S weaners on day 1 and pigs on S treatment had lower Hp concentrations on day 1 than on day 2. Enrichment treatment did not have a significant effect on ADA concentrations, but the interaction between enrichment treatment and day tended to be significant (F_2,34_ = 2.74, *P* = 0.08; Fig. 4C), with E pigs tending to have higher ADA concentrations compared to S pigs on day 1 only. There was also significant effect of day (F_2,34_ = 6.70, *P* = 0.004): ADA concentrations were significantly lower on day 4 compared to day 1 and tended to be lower on day 4 than on day 2. ADA concentrations also tended to be lower on day 2 than on day 1.

**Fig. 5:**
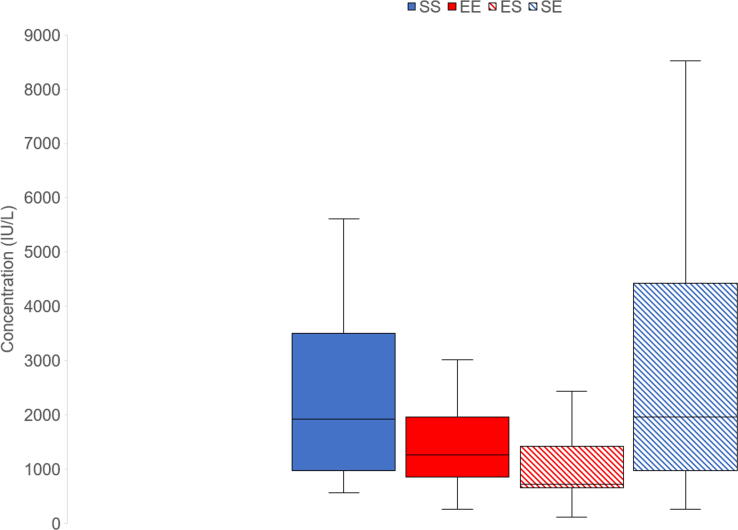
Effect of enrichment treatment during finisher stage on haptoglobin (Hp) concentrations after housing switch. Finisher pigs were kept either in the same enrichment treatment (SS and EE treatments) or were switched from enriched to standard (ES) and vice versa (SE) enrichment treatments. EE and SE finisher pigs tended (*P* < 0.1) to have decreased concentrations of Hp compared to finishers on SS and ES treatments.

In finishers, salivary cortisol concentrations were not significantly affected by either enrichment treatment during weaner/finisher stage. Neither enrichment treatment during weaner nor enrichment treatment during finisher stage significantly affected hair cortisol/cortisone concentrations measured at the end of finisher stage. There was no significant effect of the interaction between enrichment treatment during weaner and finisher stages. Alpha-amylase, Hp and ADA concentrations were not significantly affected by either enrichment treatment during weaner/finisher stage or the interaction between enrichment treatment during weaner and finisher stages. However, there was a tendency for enrichment treatment during finisher stage to affect Hp concentrations, with EE and SE pigs tending to have lower Hp concentrations than SS and ES pigs (F_1,12_ = 3.86, *P* = 0.07; Fig. 5).

## Discussion

We investigated the effects of fodder beet and jute bags as enrichment during the weaner and finisher stages of pig growth on their performance, health and stress resilience. The timing of the provision of the enrichment on these variables was a key objective of our paper. We found consequences of the enrichment and its timing on several of the investigated variables.

### Performance

The fodder beet and jute bags provided as additional enrichment during the weaner stage decreased weaner feed intake, improved weaner and finisher FCR, as well as finisher BW. There was no significant effect of enrichment treatment during weaner stage on weaner end BW. The enrichment provided only during finisher stage decreased ADFI but had no significant effect on BW and FCR.

A number of studies report positive effects of edible enrichment on feed intake (Ruiterkamp, 1987, Lyons et al., 1995, Beattie et al., 2000, van de Weerd et al., 2006, Munsterhjelm et al., 2009, Luo et al., 2020b), while others have not found any effects (Peeters et al., 2006, Averós et al., 2010, Nannoni et al., 2016, Nannoni et al., 2018, Fàbrega et al., 2019, Chou et al., 2020, Pol et al., 2021). Our ADFI results are consistent with the findings of Beattie et al. (2001), who also found decreased feed intake in E pigs. This effect may have been caused by the provision of fodder beet as enrichment in our study, and mushroom compost in the study mentioned above (Beattie et al., 2001). In both cases, it is possible that these substrates affect the feeding motivation of the pigs through their bulk properties, such as the water holding capacity of the fodder beet (Kyriazakis and Emmans, 1995). Alternatively, decreased feed intake in E pigs could have been caused by enrichment per se, i.e., pigs spent a proportion of feeding time on interacting with enrichment items.

Our results on weaner FCR are consistent with the findings of Beattie et al., 2000, van de Weerd et al., 2006. However, they contrast the results of other studies which did not find any significant effect of edible enrichment on FCR (Ruiterkamp, 1987, Lyons et al., 1995, Peeters et al., 2006, Munsterhjelm et al., 2009, Averós et al., 2010, Nannoni et al., 2016, Nannoni et al., 2018, Ipema et al., 2021, Pol et al., 2021). Organic enrichment has been found to have positive effects on pig gut microbiota, which in turn affects the way feed is utilised (Wen et al., 2021). Therefore, it is possible that root vegetables improved FCR of weaners in the present study through this mechanism. The alternative explanation may be that improved FCR in E pigs was caused by direct nutritional properties of the enrichment used.

The enrichment provided during weaner stage had long-term effects on performance, as EE and ES pigs were heavier at the end of finisher stage and had a better FCR, possibly through the mechanism suggested above. These results contrast the findings of Bolhuis et al., 2006, Luo et al., 2020b. In the study of Bolhuis et al. (2006), rooting substrates provided in previous housing did not have any significant effect on subsequent FCR. Luo et al. (2020b) found that pigs kept in enriched pens from the switch at 7 weeks of age were heavier until the end of the experiment (∼19 weeks of age), irrespective of their early life housing conditions. This means that pigs originating from barren housing, even though they had a lower BW before the housing switch, caught up after being placed in enriched pens. The converse was found in pigs from enriched housing that changed to barren pens. Bolhuis et al. (2006) as well as Luo et al. (2020b) used rooting substrates as enrichment, which might be the reason why their results differ from our findings. Different enrichment substrates and the way they are offered may affect pig performance through different routes.

### Health variables

Temporal changes in the occurrence of health variables were consistent with expectations (Chantziaras et al., 2018): in weaners, ear and body lesions were more frequent during the early stages of the phase. In finishers scouring, locomotory and respiratory variable occurrence increased with time; the same applied to the occurrence of tail lesions. However, the occurrence of ear lesions was higher during the early stages rather than later of the phase. The main effects of enrichment treatment per se were on the ear lesions of the weaners and on the body lesions of the finishers. E weaners had less ear lesions for the first two weeks postweaning and tended to have decreased occurrence of scouring. On the other hand, EE finishers had decreased occurrence of body lesions. There is significant variation in the literature about the effects of enrichment on these health outcomes, although most of the studies have the underlying expectation that enrichment will reduce the incident of tail, ear and body lesions (Lingling et al., 2018, Haigh et al., 2019, Wen et al., 2021).

To our knowledge, only four studies have addressed enrichment provision in relation to scouring in pigs. Scott et al. (2006) found no significant effect of slatted versus straw-bedded systems on clinical indicators of enteric disease. Similarly, Ipema et al. (2021) found that faecal dry matter content did not differ in pigs with and without the provision of live insect larvae as enrichment. However, bedding decreased the number of postweaning diarrhoea days in the study of Munsterhjelm (2009) and Wen et al. (2021), who found that rooting substrate in a combination with jute bags and branches improved gut microbiota. Changes in gut microbiota may also be the reason why scouring tended to occur less in E weaners in our study (Wen et al., 2021).

Several studies have addressed the effect of enrichment on tail, ear, or body lesions in pigs, as such lesions are a major welfare concern in conventional pig systems (Diana et al., 2019). However, most studies focused on the occurrence of tail and body rather than ear lesions in pigs provided with a standard versus a higher enrichment level. Lingling et al. (2018) found that toy enrichment decreased ear lesions in pigs after weaning. Chou et al. (2020) found that a cardboard cup, rubber toy, hessian cloth, and bamboo improved ear lesion score in preweaning pigs. However, Munsterhejlm (2009), Bulens (2016) and Haigh et al. (2019) found no significant effect of bedding, straw dispenser, or compressed straw block on ear lesions. Moreover, Bulens et al. (2018) detected more ear injuries in pigs provided with straw block and a hiding wall. One reason why the results are inconclusive may be the differences in methodological approaches, such as the frequency of measurements taken. Our results on reduction in the number of ear lesions are consistent with the results of studies which found positive effect of enrichment on ear lesions. This suggests that fodder beet in a combination with jute bags is effective enrichment for reducing fighting in mixed litters, as the positive effect was particularly found in 1–2 weeks postweaning.

The expected effects of enrichment on body lesions are also inconclusive. Some studies have found positive effect of a higher enrichment on body lesions (Lyons et al., 1995, Guy et al., 2002, Bulens et al., 2018, Lingling et al., 2018, Fàbrega et al., 2019, Wen et al., 2021), whereas others do not report a significant effect (van de Weerd et al., 2005, Peeters et al., 2006, Scott et al., 2006, van de Weerd et al., 2006, Munsterhjelm et al., 2009, Vanheukelom et al., 2011, Yang et al., 2018, Fàbrega et al., 2019, Pol et al., 2021), or have even found a negative effect (Munsterhjelm et al., 2009, Melotti et al., 2011, Bulens et al., 2016, Fàbrega et al., 2019, Wen et al., 2021). Because almost all these studies provided pigs with rooting substrates, we do not know why the results are inconclusive, but the reason might be again the differences in the methodological approaches. Overall, the occurrence of body lesions (particularly in weaners) was low in our study. This was probably caused by our relatively broad definition, as we gave a score of 1 only if there were more than 4 lesions found on the pig body, while one lesion was defined as at least 2 cm long scratch or wound. It is possible that the benefits of the enrichment on weaner body lesions were diminished by competition for the beet, which was occasionally observed (the competition was rarely observed in finishers). Our results suggest that fodder beet provided as enrichment improves finisher welfare, as lesions reflect fighting among pigs (Løvendahl et al., 2005). However, this effect was only present in EE pigs which also suggests the importance of providing the beet (and jute bags) during weaner as well as finisher stage.

We would like to caution about the blanket expectation on the effects of enrichment on animal health. It seems likely that different types of enrichment have different effects in the occurrence of a number of variables. Also, the underlying motivation of tail, ear and body lesions may be different and, thus, enrichment provision may have a different role in their prevention.

### Salivary and hair biomarkers

We measured concentrations of biomarkers of stress (cortisol, alpha-amylase), inflammation (Hp) and immunity (ADA) in pig saliva after weaning and after regrouping at finisher stage. Hair cortisol and cortisone concentrations were also measured at the end of weaner and finisher stages. We found that the interaction between enrichment and time (day) tended to have significant effects on weaner alpha-amylase, Hp, and ADA, as they were higher in E rather than S weaners. We also found that enrichment treatment tended to affect weaner saliva cortisol concentrations, with E weaners tending to have increased concentrations compared to S weaners. This finding supports our observations on competition for the beet, at least when it was first introduced. However, this is not consistent either with our results on decreased ear lesions in E weaners or with the results of Ko et al. 2020 who found that pigs provided with enrichment toys had decreased salivary amylase concentrations on day 1 and 2 postweaning, and decreased cortisol concentrations on day 2 (Ko et al., 2020). Pol et al. (2021) did not find any difference between S pigs and pigs which were provided with compressed algae on salivary cortisol concentrations postweaning, and Nannoni et al. (2018) did not find any significant differences in hair cortisol concentrations of pigs provided with a chain, wooden logs, or a vegetable edible block. However, sawdust, natural hemp ropes, and rubber balls were found to decrease hair cortisol concentrations in pigs (Casal et al., 2017). Further studies should be undertaken to address the significance and implications of the increased salivary cortisol concentration after introducing the enrichment in our study. So far, increases in cortisol at weaning have not been associated with other biological consequences assessed by performance, immune and inflammatory parameters (Turpin et al., 2016). It is important to note that our results may have been affected by several factors. Firstly, our piglets were constrained during the sampling on a day preweaning and on day 1 postweaning. Piglets may have perceived this handling as a stressor, e.g., attempted to flee and vocalised. Secondly, increased vocalisation in sampled animals very likely induced stress in other pigs before their actual sampling. Therefore, biomarker concentrations measured on these two days were increased more than it would have been in case of saliva sampling without any constraint. Because of the handling, the sampling procedure on a day preweaning and on day 1 postweaning took longer than in the other sampling days. Relatively high variation in sampling time could have contributed to increased individual variation among pigs.

In finishers, pigs which were provided with the enrichment after the housing switch tended to have decreased Hp concentrations that could be associated with the reduced body lesions these pigs have had, since Hp is an acute phase protein which increases when there is any inflammatory stimulus (Jain et al., 2011). Research relevant to the effect of enrichment on pig welfare after regrouping is rare, although allocation and regrouping at grower-finisher transition is common farm practice, which is stressful for pigs (Ekkel et al., 1997, Coutellier et al., 2007). Our results are consistent with the findings of de Jong et al. (1998) who did not find any significant effect of straw bedding on salivary cortisol concentrations in growing pigs after relocation (de Jong et al., 1998). Similarly, straw bedding have not been found to reduce fighting in newly mixed growing pigs (Arey and Franklin, 1995). We suggest that further research is needed to explore any potential benefits of enrichment on pig ability to cope with stress after regrouping.

## Conclusion

We investigated the consequences of provision of a novel enrichment treatment on performance, health variables, and salivary and hair biomarkers of weaner and finisher pigs kept in a slatted system. The enrichment consisting of daily provided fodder beet and jute bags, in addition to the minimal legal requirements, resulted in effects on performance both during the weaner and the finisher stage, seen mainly as improvement in FCR. Enrichment resulted in a lower number of ear lesions for two weeks postweaning, and finishers receiving the enrichment treatment throughout have had reduced body lesions.

We also investigated the effect of the timing of enrichment provision, by providing the additional enrichment during the weaner, the finisher or both stages of production. Additional enrichment had longer term effects, as finisher pigs with previous experience with the enrichment had better FCR and increased BW at the end of finisher stage. We conclude that fodder beet (in combination with jute bags) may be a promising functional enrichment for pigs, and that the timing of the enrichment provision may have long-term effects on their performance.

## Supplementary material

Supplementary data to this article can be found online at https://doi.org/10.1016/j.animal.2022.100637.

## Ethics approval

The experiment was carried out at the experimental farm of the Agri-Food and Biosciences Institute (AFBI), Hillsborough, Northern Ireland from March to July 2021. All experimental procedures, complied with the UK Animals (Scientific Procedures) Act 1986, were approved by the AFBI Animal Welfare and Ethical Review Body (AWERB) and carried out under Home Office authorization Project Licence Number PPL2851.

## Data and model availability statement

The data and models that support the study findings are available from the authors upon request.

## Author ORCIDs

**K.B.** 0002**-**4537-7341.

**R.M.** 0002**-**8139-8668.

**J.C.** 0002**-**8654-1793.

**I.K.** 0001-7703-3626.

## Author contributions

**K.B.** developed the methodology, managed the experiment, collected data, analysed data, wrote the first draft, and modified it accordingly. **R.M.** provided advice on the methodology, managed the experimental facilities, assisted with data collection on stress biomarkers and provided comments on the paper. **J.C.** provided advice on the methodology related to stress biomarkers, analysed the stress biomarkers and provided comments on the paper. **I.K.** developed the initial idea for the research, obtained and managed funding and the overall project, developed the methodology, provided advice on data analysis, and was involved in subsequent drafts of the paper.

## Declaration of interests

None.

## Acknowledgements

We would like to thank the AFBI farm staff involved in this study. We especially thank Declan Armstrong and Richard Little for trial delivery and management, and Alexandra Strain, Ryan Bradley, Ryan Murray and Peter Ffrench Mullen for assistance with data collection. Professor Sandra Edwards of Newcastle University was involved in the funding application that supported this project.

## Financial support statement

This research was part of the EU-China HealthyLivestock project. The authors wish to acknowledge that HealthyLivestock is funded by the European Union H2020 research and innovation programme under grant agreement number 773436. The European Commission’s support for the production of this publication does not constitute an endorsement of the contents, which reflect the views only of the authors, and the Commission cannot be held responsible for any use which may be made of the information contained therein.
