# Supplementary material for: Consequences of timing of organic enrichment provision on pig performance, health and stress resilience after weaning and regrouping
Source: Animal. 2022 Oct;16(10):None. doi: 10.1016/j.animal.2022.100637 (PMC9596380; doi:10.1016/j.animal.2022.100637)
Supplement: Supplementary data 1 [file mmc1.docx]

**Animal Journal:** **Consequences of timing of organic enrichment provision on pig performance, health and stress resilience after weaning and regrouping**

K. Bučková^1^, R. Muns^2^, J.J. Cerón^3^, I. Kyriazakis^1^

^1^ *Institute for Global Food Security, School of Biological Sciences, Queen's University Belfast, Belfast BT9 5DL, UK*

^2^ *Monogastric Research Group, Sustainable Agri-Food Sciences Division, Agri-Food and Bioscience Institute, Belfast, UK*

*^3^Interdisciplinary Laboratory of Clinical Analysis, Interlab-UMU, University of Murcia, 30100,* Campus de Espinardo s/n*, Murcia, Spain*

**Supplementary Material**

***Details on saliva sampling***

Saliva sampling started at 8 a.m. and was done by two (pre- and post-weaning sampling) or three (finisher sampling) experimenters. For pre-weaned piglets, an experimenter inserted a synthetic swab into the mouth of a piglet which was held outside of a pen by another experimenter. On day 1 post-weaning, pigs were sampled by inserting a swab into the mouth whilst restricted. On day 2 post-weaning, pigs chewed a swab voluntarily, although some assistance was needed (e.g., entering a pen and repeatedly present a swab to the particular pig). From day 4 post-weaning onwards, pigs were offered swabs from outside of the pens as they mostly approached and took the swabs into their mouths. In case a pig did not approach the experimenter, he/she entered a pen and presented the swab in front of the pig.

***Supplementary Tables***

Table S1. Number of excluded values for health parameters assessed in pigs during the weaner stage. All excluded values were statistical outliers.

| Parameter | Week | Number of excluded values | |
| --- | --- | --- | --- |
|  |  | Standard treatment | Enriched treatment |
| Scouring | 1-2 | 0 | 3 |
|  | 3-4 | 0 | 2 |
| Ear lesions | 1-2 | 0 | 1 |
| Body lesions | 1-2 | 0 | 1 |
|  | 5-6 | 1 | 1 |

Table S2. Number of excluded values for health parameters assessed in pigs during the finisher stage. All excluded values were statistical outliers.

| Parameter | Week | Number of excluded values | | | |
| --- | --- | --- | --- | --- | --- |
|  |  | Weaner stage | | Finisher stage | |
|  |  | Standard | Enriched | Standard | Enriched |
| Scouring | 9-11 | 1 | 1 | 1 | 1 |
| Respiratory problems | 1-2 | 1 | 0 | 0 | 1 |
|  | 6-8 | 1 | 0 | 0 | 1 |
|  | 9-11 | 0 | 1 | 0 | 1 |
| Locomotor disorders | 3-5 | 0 | 1 | 1 | 0 |
| Tail lesions | 1-2 | 1 | 1 | 1 | 1 |
|  | 6-8 | 0 | 1 | 1 | 0 |
| Ear lesions | 3-5 | 0 | 1 | 1 | 0 |
|  | 6-8 | 0 | 1 | 1 | 0 |
| Body lesions | 1-2 | 1 | 1 | 1 | 1 |
|  | 6-8 | 0 | 1 | 1 | 0 |

Table S3. Number of missing/excluded values for salivary biomarkers measured in pigs during the weaner stage.

| Biomarker | Day | Number of missing/excluded values and reason | |
| --- | --- | --- | --- |
|  |  | Standard treatment | Enriched treatment |
| Cortisol | 1 | 1 (sample was too low to measure cortisol) | 0 |
|  | 2 | 1 (statistical outlier) | 1 (missing sample) |
|  | 4 | 1 (sample was too low to measure cortisol)  1 (statistical outlier) | 0 |
| Alpha-amylase | 1 | 1 (statistical outlier) | 0 |
|  | 2 | 0 | 1 (missing sample) |
|  | 4 | 1 (statistical outlier) | 1 (statistical outlier) |
| Haptoglobin | 2 | 0 | 1 (missing sample) |
| Adenosine-  deaminase | 2 | 0 | 1 (missing sample) |

Table S4. Number of excluded values for salivary biomarkers measured in pigs during the finisher stage. All excluded values were statistical outliers.

| Biomarker | Day | Number of excluded values | | | |
| --- | --- | --- | --- | --- | --- |
|  |  | Weaner stage | | Finisher stage | |
|  |  | Standard | Enriched | Standard | Enriched |
| Cortisol | 1 | 1 | 1 | 0 | 3 |
|  | 2 | 0 | 2 | 1 | 0 |
|  | 4 | 0 | 3 | 0 | 3 |
| Alpha-amylase | 1 | 3 | 1 | 1 | 3 |
|  | 4 | 0 | 2 | 1 | 1 |
| Haptoglobin | 1 | 1 | 1 | 1 | 0 |
|  | 2 | 1 | 0 | 0 | 2 |
|  | 4 | 1 | 2 | 1 | 2 |
| Adenosine-  deaminase | 1 | 1 | 0 | 1 | 0 |
|  | 2 | 1 | 0 | 0 | 1 |

***Supplementary Figures***


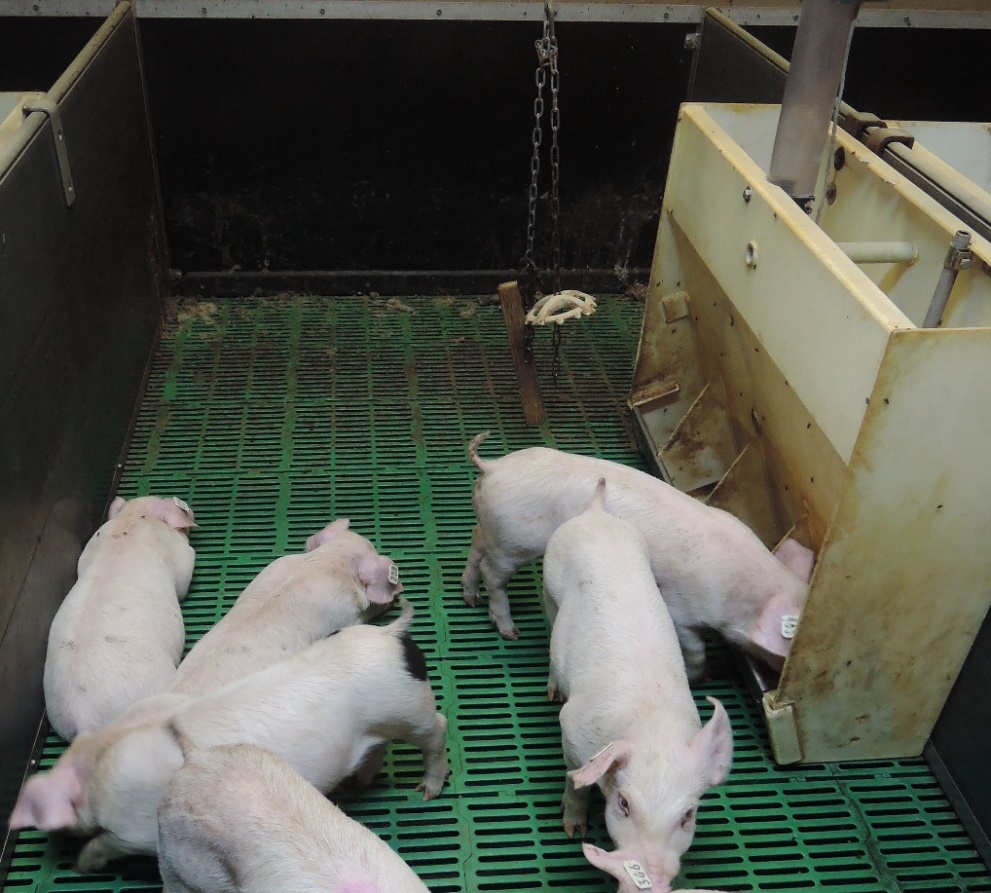


Figure S1. The set up during the pig weaner stage of the experiment. Standard enrichment housed weaner pigs had access to a plastic toy and a piece of softwood hanging in the pen as enrichment.


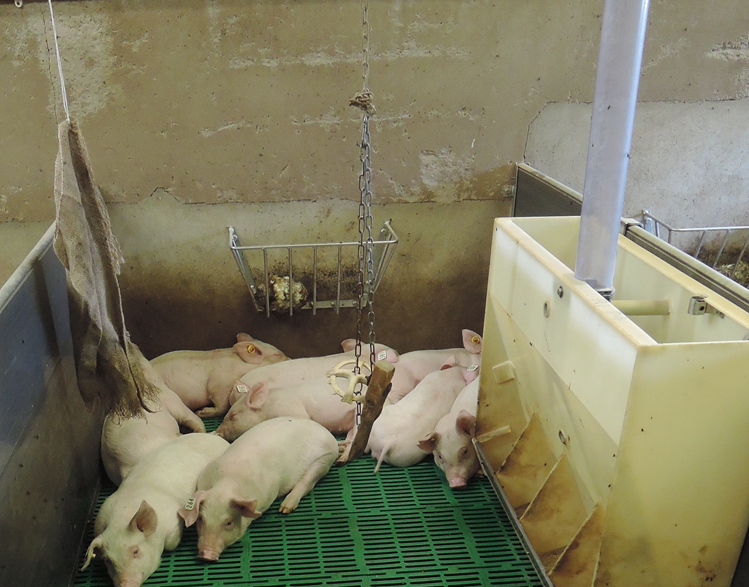


Figure S2: The set up during the pig weaner stage of the experiment. Enriched-housed weaner pigs had access to a jute bag and fodder beet in a rack as additional enrichment.


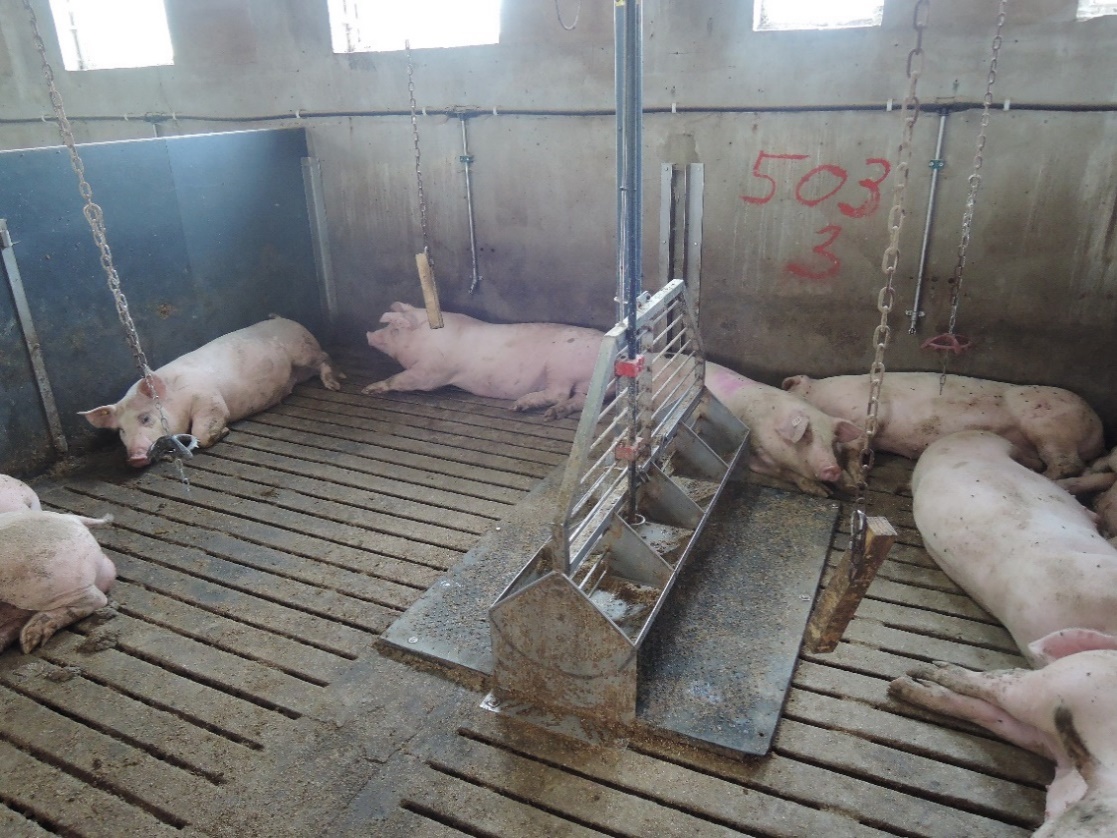


Figure S3. The set up during the pig finisher stage of the experiment. Standard enrichment housed finisher pigs had two plastic toys and two pieces of softwood as enrichment.


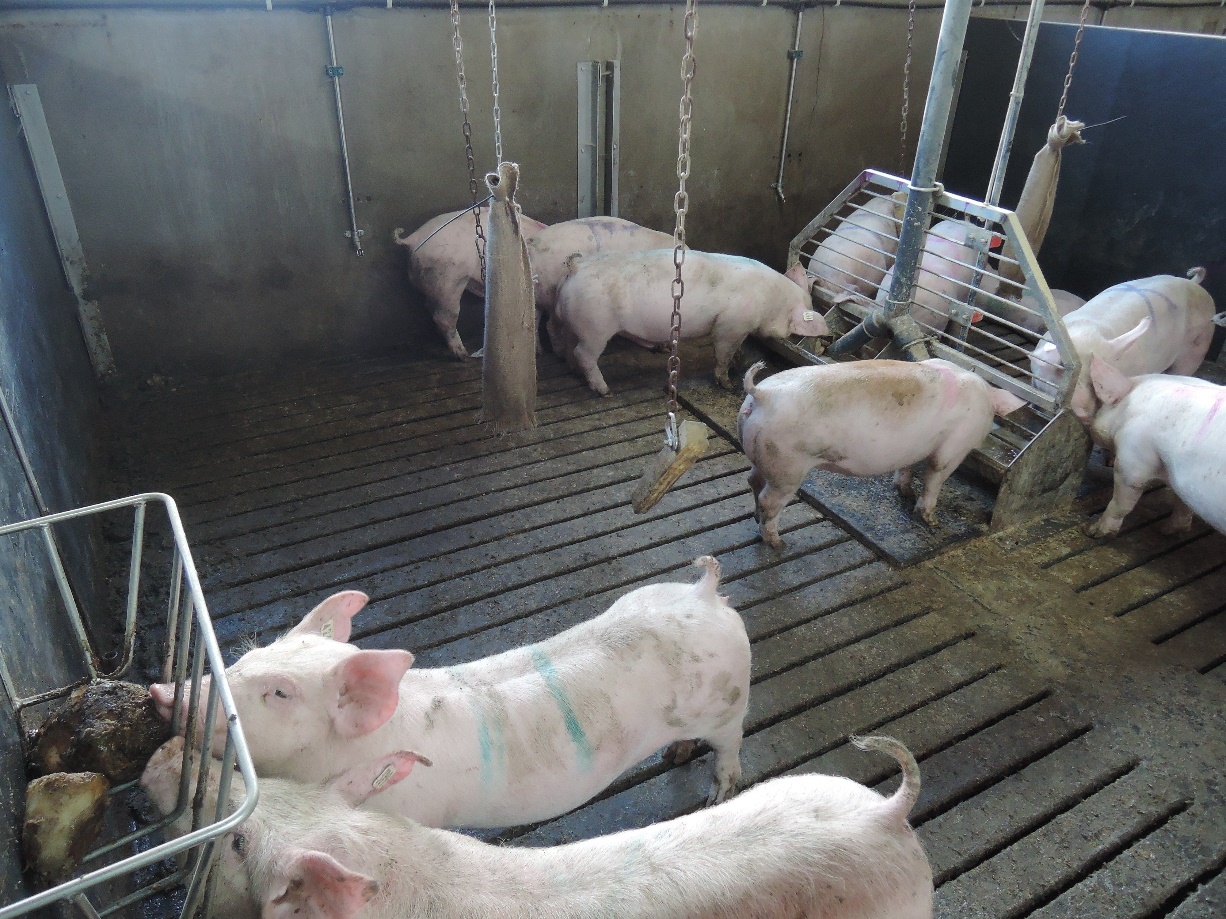


Figure S4: The set up during the pig finisher stage of the experiment. Enriched-housed finisher pigs had access to two jute bags and fodder beet in a rack as additional enrichment.
